# Training characteristics of professional development activities in elite football players: a systematic review

**DOI:** 10.3389/fpsyg.2026.1826135

**Published:** 2026-05-15

**Authors:** Miao Shen, Sihang Wang, Guanjie Wang, Jiejin Zhou, Pei Li

**Affiliations:** School of Physical Education and Sport Science, South China Normal University, Guangzhou, China

**Keywords:** developmental milestones, football-specific training, multi-sport participation, perceptual-cognitive skills, talent development

## Abstract

**Objective:**

To systematically review the literature to investigate the developmental training characteristics associated with progression in professional football.

**Design:**

Systematic review.

**Methods:**

Comprehensive search of three electronic databases: Web of Science, PubMed, and Scopus up to August 2025. Studies were included if they reported empirical data on milestone events, accumulated training hours, training structure, subjective perceptions, and multi-sport participation among players of varying competitive skill levels.

**Results:**

Forty-two studies met inclusion criteria. Findings indicated that higher-level players typically initiated football involvement and reached professional academies at significantly younger ages than lower-level counterparts. In terms of training structure, successful career trajectories commonly followed a specific pattern where informal play predominated during childhood, progressively giving way to intensive coach-led practice throughout adolescence. Elite female players distinctively utilized mixed-gender activities to compensate for limited structured opportunities. Regarding perceptual factors, elite players reported markedly higher perceptions of effort and challenge during training activities. Furthermore, they demonstrated superior decision-making accuracy and pattern-recognition skills compared with non-elite athletes. Finally, data showed that most elite players engaged broadly in multiple sports during childhood, particularly invasion team games, before specializing.

**Conclusion:**

This review provides evidence that football-specific activities, particularly accumulated hours and subjective perceptions, play a critical role in shaping professional pathways. However, inconsistent effects regarding milestones and limited female-specific evidence caution against overgeneralization. Future research is required to prioritize longitudinal designs, gender-comparative analyses, and cross-cultural perspectives to refine recommendations for the optimization of athlete development systems.

**Systematic review registration:**

Open Science Framework (DOI: 10.17605/OSFIO/QNKSF).

## 1 Introduction

Football is widely recognized as a highly competitive sport, requiring athletes to demonstrate advanced technical skills, tactical awareness, physical conditioning, and psychological adaptability (Afonso et al., 2025). Despite its massive global participation base of approximately 265 million players, only 0.04% eventually reach professional leagues (Sweeney et al., 2021). This striking disparity underscores the difficulty of the professionalization pathway and highlights the need to identify modifiable factors that facilitate the transition from youth to elite levels.

To navigate this highly selective pathway, scholars have extensively investigated optimal approaches to talent identification (TID) and talent development (TDE). Traditional models have predominantly focused on early TID, often relying on exclusionary cross-sectional physical or technical assessments (Williams and Reilly, 2000). However, such early identification mechanisms are highly susceptible to maturational differences, frequently leading to systematic selection biases and premature talent dropout (Burgess and Naughton, 2010). Consequently, contemporary academic consensus has shifted toward the understanding that dynamic TDE is inherently more critical than static talent identification. According to the Differentiated Model of Giftedness and Talent (DMGT), innate giftedness merely serves as a baseline; the provision of optimal developmental environments and systematic practice are the decisive variables that transform natural gifts into elite athletic talent (Vaeyens et al., 2008).

Throughout the protracted process of talent development, professional achievement is influenced by a multitude of factors spanning genetic, physiological, psychological, and socio-cultural dimensions. Previous research has identified the birthplace effect (MacDonald et al., 2009), the relative age effect (Morganti et al., 2024), and social support from parents and coaches (Baxter-Jones and Maffulli, 2003) as significant determinants of career trajectories. Furthermore, elite players consistently exhibit distinct psychosocial characteristics, such as superior self-regulation and resilience, which are critical for navigating the pathway to excellence (Gledhill et al., 2017). While these factors provide valuable insights, they are largely innate, genetically predisposed, or dictated by macro-environmental contexts (e.g., birth month or city size), making them inherently difficult to modify through targeted daily interventions.

In contrast, post-developmental training activities constitute the most operative and highly controllable domain within athlete development. These modifiable factors encompass the timing of developmental milestones (Ford et al., 2012; Roca et al., 2012), accumulated training hours (Coutinho et al., 2023; Hornig et al., 2016), training structure (Machado et al., 2020; Machado et al., 2024a), subjective perceptions of training (e.g., challenge, enjoyment, motivation) (Andrew et al., 2024; Berry et al., 2008), and engagement in other sports (Andrew et al., 2022; Sieghartsleitner et al., 2018). Exploring the efficacy of these variables requires the foundational support of two influential theoretical frameworks. The Deliberate Practice Theory (DPT) posits that expert performance is achieved through sustained, goal-oriented, and effortful training accumulated over many years (Ericsson et al., 1993). However, critics argue that blindly applying the “10,000-h rule” within team sports can lead to premature specialization, significantly increasing the risk of overuse injuries and motivational burnout among young athletes (Burgess and Naughton, 2010; Güllich et al., 2022).

Complementing and refining this perspective, the Developmental Model of Sport Participation (DMSP) proposes a staged developmental pathway (Côté and Vierimaa, 2014). The DMSP underscores the developmental benefits of early diversification and “deliberate play,” suggesting that delayed specialization not only enhances athletes’ adaptability but is also positively associated with sustained elite performance in adulthood (Sieghartsleitner et al., 2018; Taylor et al., 2024). However, a stark contradiction exists between these theoretical recommendations and the applied realities of modern football. Driven by the hyper-competitive nature of youth academies, practitioners often face immense systemic pressure to identify and specialize talent as early as possible, fearing that delayed football-specific investment might leave players trailing behind their peers. This creates a profound developmental paradox: while scientific evidence warns against the physical and psychological toll of premature specialization, selection systems frequently reward early specialized proficiency. Consequently, striking an appropriate balance between early specialization and diversified sport participation—and understanding how elite players historically navigated this conflict—has become a central issue in constructing optimal talent development systems.

Unravelling how successful athletes actually navigated these conflicting developmental pathways requires a detailed, longitudinal examination of their accumulated training histories. However, despite the increasing scholarly focus on football training and development, recent comprehensive analyses, such as the systematic review by Verbeek et al. (2025), reveal that the vast majority of literature on football talent development remains confined to “static-inter-individual” designs. These isolated studies often lack the systematic, quantitative integration of specific training structures and progression patterns needed to map a complete developmental trajectory. To address this critical gap, the present review systematically synthesizes empirical evidence on training activities within the developmental pathways of professional football players, focusing on four key areas: (1) the timing of developmental milestones; (2) the duration and structural composition of training across stages; (3) the subjective perceptions of training (e.g., enjoyment, effort, challenge); and (4) patterns of participation in other sports. By integrating existing evidence, this review seeks to provide concrete answers regarding which training patterns constitute the optimal developmental environment, thereby offering evidence-based, practical recommendations for optimizing youth football development systems and career pathways.

## 2 Method

### 2.1 Search Strategy

The review protocol was prospectively registered with the Open Science Framework (DOI 10.17605/OSF.IO/QNKSF), and the review process was conducted in accordance with the Preferred Reporting Items for Systematic Reviews and Meta-Analyses (PRISMA) guidelines (Moher et al., 2009). A systematic literature search was performed on the Web of Science, PubMed and Scopus databases from inception to 16th August 2025. The search strategy employed keywords including the population: “football or soccer”, “athlete or player”, “professional or elite”, “woman or girl or female”. Training variables: “developmental activity” or “development” or “talent development” or “milestone” or “training hour” or “training structure” or “practice” or “play” or “competition”, “rating” or “perception” or “deliberate practice” or “deliberate play” or “DMSP” or “specialization” or “diversification” or “multi-sport” or “multisport”. Each of these keywords was first carried out independently and then combined into a Boolean search using the AND operator. In addition, reference lists from included articles and relevant reviews were manually examined for further eligible studies.

### 2.2 Inclusion and exclusion criteria

The selection of each article was carried out independently by two authors (JZ, GW). In case of disagreement, the selection of articles was judged by more experienced experts (SW, PL) until all authors were in agreement. Inclusion criteria were as follows:(1)Studies addressing training activities in the developmental pathways of football players, including but not limited to milestone events (e.g., initiation age, entry into academy or professional systems), accumulated training hours, training structure (practice, play and competition), rating and perceptions of training (e.g., enjoyment, motivation, effort, challenge) and engagement in other sports. (2) Studies involving male or female football players who had undergone systematic training at academy, varsity, professional, or national levels. (3) Publications written in English and published in peer-reviewed journals. Exclusion criteria were as follows: (1) Studies focusing solely on non-training-related determinants of career development, such as relative age effect, birthplace effect, or social support. (2) Studies examining sports other than football without football-specific subgroup analyses. (3) non-peer-reviewed sources, including conference abstracts.

### 2.3 Data extraction

The following data were extracted for each included study: the first author and publication year; the study sample; the developmental milestone events (including age at initiation of football; age at organized training; age at first formal competition; age at entry into youth academy; age at professional debut; age at national team selection; and age at mixed-gender competition exposure); cumulative training hours; training structure; training ratings and perceptual differences; involvement in other sports; and key findings.

### 2.4 Assessment of methodological quality

Articles were independently appraised for methodological quality by two reviewers (SW, PL). For 39 cross-sectional studies, the Appraisal Tool for Cross-Sectional Studies (AXIS) (Downes et al., 2016) was employed. The AXIS checklist consists of 20 items grouped into three domains: reporting quality (7 items), study design (7 items), and potential sources of bias (6 items). The instrument was chosen for its suitability in evaluating observational and cross-sectional designs, which predominate in research on athlete developmental activities, and has been applied in previous systematic reviews within sports science. All 20 items were retained to capture methodological limitations relevant to this field, including non-response bias and confounding, which are particularly pertinent given the retrospective designs of many included studies. Each item was rated dichotomously as “yes” (1 point) if the criterion was fulfilled, “no” (0 points) if not fulfilled, or “not applicable,” which was excluded from the denominator. Total quality scores therefore ranged from 0 to 20. For 3 longitudinal cohort studies, the Newcastle–Ottawa Scale (NOS) was applied, which evaluates three domains: selection of cohorts, comparability, and outcome assessment (Wells et al., 2014). This tool was selected due to its established use in assessing prospective and retrospective cohort designs, enabling a more accurate appraisal of follow-up adequacy, representativeness, and confounding control. Each study could receive up to nine stars, with higher scores indicating higher methodological quality. Discrepancies in ratings between the two reviewers were resolved through discussion until consensus was reached. Consistent with prior methodological reviews, studies were not excluded on the basis of quality scores. Instead, the appraisal was used to highlight methodological strengths and weaknesses across the body of evidence, thereby informing the interpretation of results rather than restricting the dataset. Subgroup analyses by study quality were not performed.

## 3 Results

**Figure 1:**
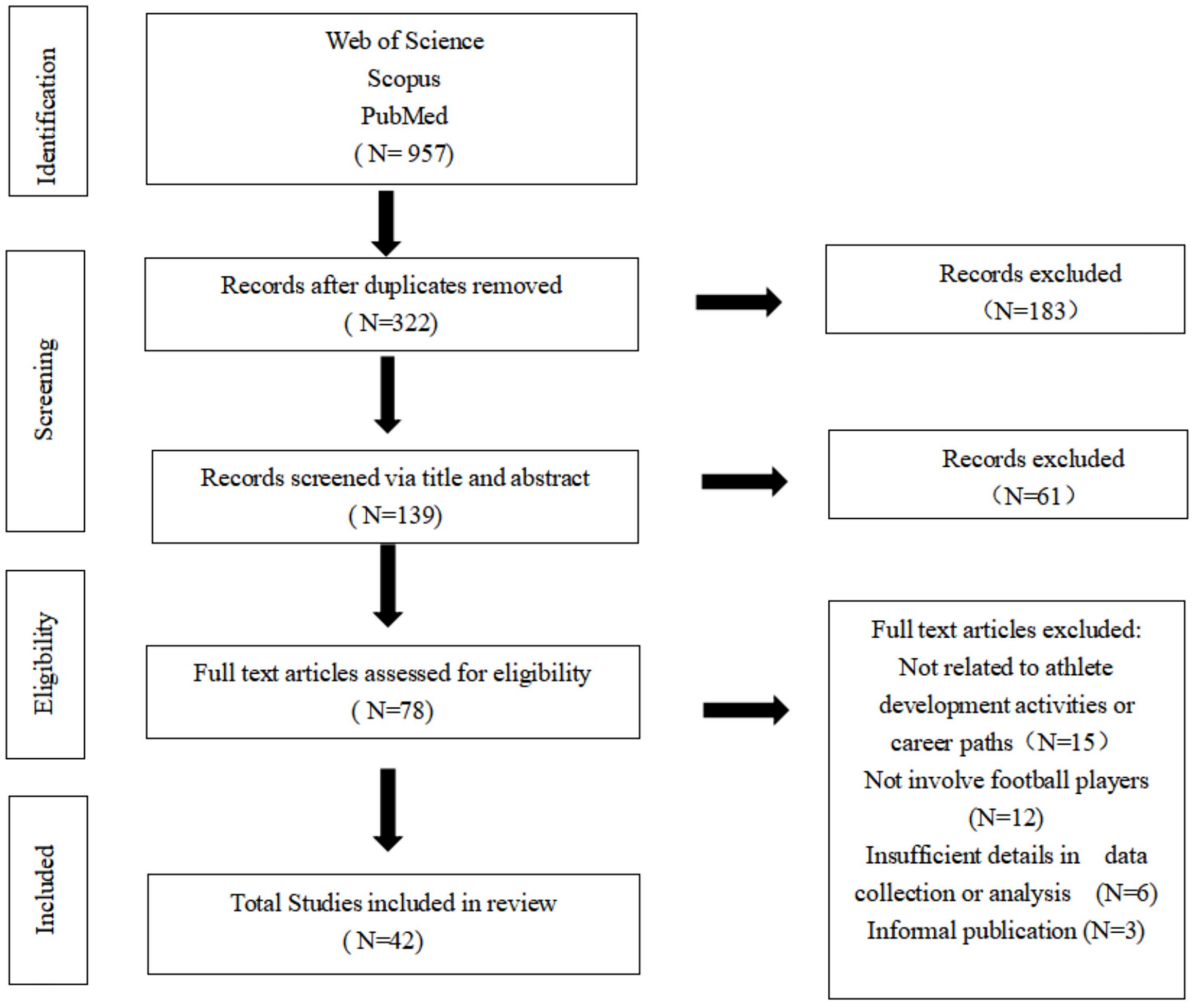
PRISMA flow diagram showing the literature search, screening, and eligibility results.

The search results are illustrated in the PRISMA flow diagram. The systematic search initially identified 957 records. After removing duplicates and screening titles, 322 articles remained. Screening of abstracts further reduced the number to 139. Finally, 42 studies fulfilled all inclusion criteria and were retained for full analysis (Figure 1).

### 3.1 Descriptive results

A total of 42 studies were included in this review, spanning the period from 1988 to 2024. The temporal distribution of publications exhibited a gradual increase over time. The earliest study was published in 1988, whereas the most recent appeared in 2024. The year 2009 represented the peak in publication output, with five studies. Broadly, two distinct phases can be delineated: an initial period of sporadic publications from 1988 to 2007, characterized by relatively few contributions per year; and a more prolific phase from 2008 onward, during which the majority of studies were published, reflecting the burgeoning academic interest in athlete developmental activities. Geographically, the studies originated from 16 countries, with the majority concentrated in Europe (*n* = 36, 85.7%). The United Kingdom contributed the most (*n* = 10, 23.8%), followed by Germany (*n* = 7, 16.7%) and Switzerland (*n* = 3, 7.1%). Methodologically, the majority (*n* = 39, 92.9%) employed cross-sectional designs, utilizing retrospective questionnaires (*n* = 35, 83.3%) or interviews (*n* = 3, 7.1%) as primary data collection methods, supplemented by experimental tasks (*n* = 5, 11.9%) in a subset. Only three studies (7.1%) adopted longitudinal cohort designs, tracking participants over 2–10 years via repeated questionnaires and performance assessments, thereby providing stronger causal insights into skill trajectories. Sample characteristics emphasized male-dominated cohorts, with 36 studies (85.7%) focusing exclusively on male athletes, primarily in football/soccer (*n* = 24, 57.1%). Female-only samples were limited to six studies (14.3%). Athlete levels varied widely, encompassing national-level (*n* = 14, 33.3%), professional-level (*n* = 28, 66.7%), youth academy (*n* = 10, 23.8%), varsity (*n* = 1, 2.4%), and amateur (*n* = 10, 23.8%) participants.

### 3.2 Methodology quality

The Critical Appraisal scores from both reviewers were compared, and Cohen’s kappa coefficient (*κ*) analysis was conducted to determine the level of agreement. Kappa analysis revealed almost perfect agreement between raters when using the NOS tool (κ = 0.92) and substantial agreement when using the AXIS tool for critical appraisal (κ = 0.87). The 39 cross-sectional studies demonstrated moderate to high methodological quality, with total AXIS scores ranging from 14 to 20; notably, 32 studies (82.1%) achieved scores ≥16, indicating high quality, while the remaining seven studies scored 14–15 (moderate quality). Common weaknesses across studies included the lack of statistical power calculations or justification of sample size and insufficient reporting on non-responders and potential non-response bias. These issues reflect recurring limitations in retrospective, questionnaire-based research designs that dominate the literature in this area. The three longitudinal cohort studies were appraised using the NOS tool. All three achieved 8 out of 9 stars, reflecting high methodological quality. The cohorts were broadly representative of elite youth football populations, follow-up periods were sufficiently long to capture relevant outcomes, and outcome measures were based on objective records.

## 4 Discussion

### 4.1 Milestone events

In athletes’ career progression, milestone events represent key transitional time points that mark critical shifts in their developmental pathways (Ford and Williams, 2008). These events not only provide a clear temporal framework for understanding the trajectory of athletes’ career development but also play a pivotal role in determining whether they attain higher competitive levels (Gissis et al., 2006).

Milestones typically encompass the staged shift from initial recreational engagement to domain-specific investment, including first exposure to football, initiation of organized training, engagement in formal competitions, entry into youth academies, first-team debut and selection for national teams. For clarity, milestone evidence was organized into early developmental milestones (Table 1), including football initiation, supervised training, and formal competition, and advanced pathway milestones (Table 2), including academy entry, professional-team involvement, and national-team selection.

**Table 1:** Early developmental milestones in football players.

Author	Sample	Start age	Supervised Training	Formal competition
Roca et al. (2012)	64 males	High:5.2 ± 1.3 Low:5.6 ± 1.1 Rec:5.5 ± 1.2 (NS)	High:7.5 ± 1.9 Low:8.0 ± 2.2 Rec:7.9 ± 1.9 (NS)	High:8.5 ± 2.0 Low: 8.9 ± 1.9 Rec: 9.3 ± 1.3 (NS)
Hendry et al. (2019)	45 females	All:4.95 ± 1.64 NT:5.43 ± 2.06 Va:4.50 ± 0.96 (NS)	All 5.65 ± 1.85 NT 6.28 ± 2.19 Va 5.05 ± 1.21 (NS)	
Güllich (2019)	29 females	NT: 4.4 ± 2.3 BL: 6.1 ± 1.4 (*)	NT: 7.1 ± 3.8 BL: 5.7 ± 1.5 (NS)	NT:9.2 ± 3.7 BL:6.7 ± 1.4 (*)
Hendry and Hodges (2018)	128 males	Academy Only:5.42 ± 1.63 Youth Pro:5.08 ± 1.35 Youth Pro Only:5.1 ± 1.51 Adult Pro:4.89 ± 1.65 (NS)		
Machado et al. (2024a)	131 males	Brazil: 6.2 ± 2.6 Spain: 4.6 ± 1.4 (***)	Brazil:8.4 ± 2.6 Spain: 5.8 ± 2.1 (***)	Brazil:8.8 ± 2.2 10.5 ± 2.0 (11 side) Spain: 6.1 ± 1.8 11.4 ± 1.3 (11 side) (***)
Hornig et al. (2016)	102 males	NT + 1stBL: 4.3 ± 1.1 NT: 4.1 ± 0.5 1stBL:4.4 ± 1.3 AM: 4.4 ± 1.1 (NS)	NT + 1stBL: 6.0 ± 2.0 NT: 5.3 ± 1.1 1stBL:6.4 ± 2.2 AM: 5.6 ± 2.0 (NS)	NT + 1stBL: 7.4 ± 2.8 NT: 6.6 ± 1.1 1stBL:7.8 ± 3.2 AM: 7.5 ± 2.5 (NS)
Andrew et al. (2024)	56 females	Pro:5.9 ± 1.7 EA: 7.0 ± 2.3 (*)	Pro:6.8 ± 2.0 EA:8.8 ± 2.7 (*)	Pro:8.6 ± 3.2 EA: 9.5 ± 2.6 (NS)
Ford and Williams (2012)	32 youth males	Pro: 4.2 ± 1.2 Non-Pro: 5.1 ± 1.8 (NS)	Pro: 5.9 ± 1.1 Non-Pro: 6.8 ± 1.3 (*)	Pro: 7.3 ± 1.2 Non-Pro: 7.8 ± 1.4 (NS)
Ford et al. (2012)	328 males	All:4.88 ± 1.413 England, Portugal, Sweden < France, Ghana, Mexico (*)	All: 6.93 ± 1.82 Group diff (NS)	All:8.65 ± 1.95 Group diff (NS)
Ford et al. (2009)	33 males	Pro:5.3 ± 2.1 EA:5.5 ± 1.8 (NS)		
Haugaasen et al. (2014)	745 youth males	Pro: 5.3 ± 1.2 Non-Pro: 5.4 ± 1.4 (NS)		
Berry et al. (2008)	32 males	Expert: 8.3 ± 1.9 Less skilled: 8.4 ± 2.0 (NS)		
Andrew et al. (2022)	25 males	Skilled: 6.5 ± 2.3 Less-skilled:9 ± 2.9 (*)	Skilled: 8.9 ± 2.3 Less-skilled:10.8 ± 3.4 (NS)	Skilled: 10.8 ± 2.1 Less-skilled:12.5 ± 2.6 (NS)
Charbonnet et al. (2024)	84 females		C1:13.62 ± 4.24 C2: 8.62 ± 2.46 C3: 7.94 ± 2.25 (*)	
Zibung and Conzelmann (2013)	346 males		All: 6.07 ± 1.31 C1: 5.40 ± 0.75 C2: 7.44 ± 1.05 C3: 5.24 ± 0.94 C4: 6.21 ± 1.18 C5: 5.48 ± 0.90 (NS)	
Coutinho et al. (2023)	546 youth males	Football: 5.15 ± 1.74 Basketball:5.47 ± 2.2 Handball: 5.28 ± 1.93 Volleyball:5.36 ± 2.4 Water Polo:5.05 ± 2.25		
Leite et al. (2009)	112 males	6–10 (92.9%) 11–14 (7.1%)		
Toering et al. (2009)	444 males	Elite: 5.9 ± 1.7 Non-elite: 6.1 ± 2.4 (NS)		
Huijgen (2013)	131 males	Pro:6.5 ± 1.4 AM:6.9 ± 1.8		
Gissis et al. (2006)	54 youth males	Elite:11.3 Sub elite:12.8 Rec: 13.5		
Christensen and Sørensen (2009)	25 males	5–7		
Helsen et al. (1998)	73 males	5.5 ± 0.3	7.1 ± 0.3	
Ford and Williams (2008)	20 males	Football-No Gaelic: 6.2 ± 1.5 Football-Gaelic: 6.6 ± 2.1 (NS)	Football-No Gaelic: 7.9 ± 1.1 Football-Gaelic: 8.7 ± 1.6 (NS)	Football-No Gaelic: 8.6 ± 0.7 Football-Gaelic: 9.3 ± 1.8 (NS)
Ward et al. (2007)	203 males	Elite: 5.4 ± 1.5 Sub elite: 5.0 ± 1.2 (NS)	Elite: 6.5 ± 2.0 Sub elite: 7.6 ± 1.8 (*)	Elite: 6.6 ± 1.8 Sub elite: 7.9 ± 2.2 (*)
Hendry et al. (2014)	148 youth males	U13:4.6 ± 1.4 U15:5.6 ± 1.5 U17:5.4 ± 1.6 (*)	U13:5.6 ± 1.7 U15:6.4 ± 1.8 U17:6.6 ± 1.8 (*)	
Sieghartsleitner et al. (2018)	294 males		Overall:6.3 ± 1.3 Cluster 1–5: (5.4–7.9) (NS)	
Güllich et al. (2017)	44 males		Strong: 4.5 ± 1.4 Weak: 4.4 ± 1.8 (NS)	Strong:5.9 ± 2.1 Weak:5.5 ± 1.8 (NS)
Güllich et al. (2020)	100 youth males		5.6 ± 1.6	5.9 ± 1.6

**Table 2:** Advanced pathway milestones in football players.

Author	Sample	Football Academy	Professional team	National team
Roca et al. (2012)	64 males	High:12.2 ± 2.6 Low:11.0 ± 3.9 (NS)	High:17.6 ± 1.7 Low:17.6 ± 1.2 (NS)	
Hendry et al. (2019)	45 females	All:14.03 ± 3.90 NT:15.42 ± 2.98 Va:12.14 ± 4.31 (NS)		Youth National: All:14.85 ± 3.29 NT:15.65 ± 1.5 Va:13.5 ± 4.89 (NS)
Güllich (2019)	29 females		NT:17.9 ± 2.7 BL:17.5 ± 0.9 (NS)	Senior National: 19.6 ± 1.5
Hendry and Hodges (2018)	128 males	Academy Only:11.39 ± 2.09 (**) Youth Pro:9.53 ± 2.18 Youth Pro Only:9.59 ± 2.20 Adult Pro: 9.44 ± 2.24		
Hornig et al. (2016)	102 males		NT + 1stBL: 21.8 ± 3.1 NT: 20.4 ± 3.3 (*) 1stBL:22.6 ± 2.8 (*)	NT + 1stBL: 14.1 ± 2.3 NT: 14.0 ± 2.1(**) 1stBL:14.2 ± 2.4(**) AM: 16.6 ± 2.1(**) Senior NT: 22.7 ± 2.3 (NS)
Andrew et al. (2024)	56 females	Pro:12.4 ± 2.8 EA: 13.7 ± 1.9 (NS)		
Ford and Williams (2012)	32 youth males	Pro: 10.4 ± 2.7 Non-Pro: 10.8 ± 1.7 (NS)		
Ford et al. (2012)	328 males	All:11.95 ± 2.56 Portugal, 8.3, England 10.1 < others (*)		
Ford et al. (2009)	33 males	Pro: 9.5 ± 2.7 EA: 8.8 ± 2.4 (NS)		
Haugaasen et al. (2014)	745 youth males			
Berry et al. (2008)	32 males		Expert: 18.4 ± 1.6 Less-skilled: 19.0 ± 1.3 (NS)	
Andrew et al. (2022)	25 males	Skilled: 10.9 ± 3.2 Less-skilled:10.6 ± 2.2 (NS)		
Christensen and Sørensen (2009)	25 males	13–14		
Helsen et al. (1998)	73 males			Youth NT: 9.9 ± 1.9
Ford and Williams (2008)	20 males		Football-No Gaelic: 17.3 ± 0.7 Football-Gaelic: 18.1 ± 1.5 (NS)	
Schroepf and Lames (2018)	636 youth males			Youth NT: later Senior NT players first entered the Youth NT pathway at U16 (~25%), U17 (~20%), U18 (~20%). Senior NT: all later Senior NT players had ≥1 Youth NT appearance.
Güllich (2014)	3,103 males	10–19 Turnover rate: 24.5%		15–19 Turnover rate: 41%

High = high-level; Low = low-level; Rec = recreational; NT = national team; Va = varsity; BL = Bundesliga; 1stBL = first Bundesliga; Pro = professional; Non-Pro = non-professional; EA = ex-academy; AM = amateur; C1-C5 = cohort, cluster, or developmental-stage groups as defined in the original studies; NS = non-significant. (*, **, ***) = significant differences at *p* < 0.05, *p* < 0.01, and *p* < 0.001, respectively.

Current research indicates that the start age of male professional football players in these events at approximately 4–6 years (first contact) (Ford et al., 2012; Hornig et al., 2016), 6–7 year (organized training) (Hendry et al., 2019; Roca et al., 2012), 7–9 year (official competition) (Güllich, 2019; Machado et al., 2024a), 9–12 year (academy entry) (Hendry and Hodges, 2018; Ford et al., 2009), 17–19 year (senior debut) (Berry et al., 2008; Güllich, 2019), 14–16 year (youth national team) (Hendry et al., 2019; Hornig et al., 2016), and 18–22 year (senior national team) (Güllich, 2019; Hornig et al., 2016). Female football players exhibit similar career milestones to their male counterparts, although some studies suggest that females enter youth academies at a slightly later age. For example, Andrew et al. (2024) reported a mean age of entry into youth academies of 12.4 years for professional English female football players, whereas Hendry et al. (2019) found a mean entry age of 15.4 years for Canadian national-level female players, both notably later than typical male cohorts. However, due to the paucity of systematic gender-comparative studies, the significance of these differences remains to be substantiated. Additionally, some studies have examined athletes’ peak performance ages (Ford and Williams, 2011), and the initial age participate in mixed-gender activities (Andrew et al., 2024; Güllich, 2019; Hendry et al., 2019). For instance, previous research indicates that the average age of athletes winning the UEFA Ballon d’ Or and FIFA World Player of the Year awards is 25.6 years (Ford and Williams, 2011). This data reflect the complete cycle from initiation to maturity.

Across competitive tiers, higher-level players often exhibit earlier timing on several milestones (Andrew et al., 2022; Ford and Williams, 2011, 2012; Güllich, 2019; Hendry and Hodges, 2018; Hornig et al., 2016; Ward et al., 2007). For instance, Güllich (2019) found that German national-level athletes initiated football exposure significantly earlier (4.4 years) than Bundesliga-level athletes (6.1 years). Similarly, national-level athletes joined professional teams at a younger mean age (20.4 years) compared to their professional counterparts (22.6 years) (Hornig et al., 2016). However, such level-related differences are not invariably significant. Moreover, several studies suggest that the timing of milestones does not consistently predict career success (Berry et al., 2008; Ford et al., 2009; Ford and Williams, 2012; Güllich et al., 2017; Haugaasen et al., 2014; Hendry et al., 2019; Roca et al., 2012; Toering et al., 2009; Zibung and Conzelmann, 2013), implying that other factors, such as training quality, psychological attributes, and environmental support, may exert substantial influence on developmental outcomes.

Milestone timing also varies across nationalities. In a seven-nation comparison, England and Portugal tended to show earlier football initiation and academy entry than France, Ghana, and Mexico (Ford et al., 2012). More recently, Machado et al. (2024a) contrasted Brazil and Spain, noting earlier initiation, structured practice, and competition ages in Spain but later onset of 11-a-side play relative to Brazil. These disparities may be closely linked to national variations in football infrastructure accessibility, program prevalence, cultural preferences, and developmental pathway orientations.

Milestone events offer a valuable lens for understanding athletes’ career development, yet the association between their timing and competitive success is modulated by multiple factors, including gender, performance level, and regional differences. Existing studies highlight a general trend toward earlier milestones among higher-level athletes, although this pattern does not serve as a universal predictor, and cross-national variations further complicate the regularity of developmental pathways. Future research should prioritize systematic gender comparisons, deeper explorations of environmental influences on milestone timing, and longitudinal tracking data to elucidate the multidimensional determinants of career success. Such endeavors will contribute to the construction of more comprehensive models of athletes’ career development, thereby informing the optimization of youth training systems and policy formulation.

### 4.2 Football-specific training hours

Football-specific accumulated engagement constitutes a core factor in the career development of football players, encompassing various forms of activities including organized coach-led practice, play, and competition, with some studies also incorporating individual practice (Andrew et al., 2022). In the present review, football-specific activities are considered from two complementary perspectives: accumulated training hours across developmental stages (Table 3) and the structural composition of training activities (Table 4). The participation duration in football is typically categorized by developmental stages, with common structures including childhood, early adolescent, and late adolescent, though specific age ranges may vary (Andrew et al., 2024; Hendry and Hodges, 2018; Machado et al., 2024a). This phased approach facilitates identifying shifts in training priorities and pinpointing peak training periods during critical developmental phases.

**Table 3:** Accumulated training hours in football-specific activities across developmental stages.

Author	Sample	Childhood Hrs	Adolescent Hrs	Total *Cum*. hrs
Roca et al. (2012)	64 males	Total/h: High 449 > Low 317 > Rec 190 (*) Play: High 339 > Low 208 > Rec 142 (*) Play 230 > Prac 54 > Comp 35 (*)	Total/h: High 467 = Low 390 > Rec 223(*) Play: High 195 > Rec 139(*) Prac: High 178, Low 137 > Rec 48(*) Comp: High 94, Low 91 > Rec 37(*) Play 165 > Prac 121 > Comp 74 (*)	High 5,947 > Low 4,564 > Rec 2,670 (*)
Hendry et al. (2019)	45 females		Prac: Adolescent>Childhood Diff = 2,168(*) Comp: Adolescent>Childhood Diff = 204.9 (*)	NT 8361.6 > Va 6,369(NS) Play (*Cum* ≤ 19 yrs): NT > Va Diff = 519.3 (*)
Güllich (2019)	29 females	Prac 7–10 y (h/wk): BL 3.0 > NT 2.3 (*)	Prac 11–14 y (h/wk): BL 4.7 > NT 3.6 (*)	
Hendry and Hodges (2018)	128 males	Prac: Pro (Youth + Adult) 1,529–1,549 > Academy-only 1,222 (*) Play: Adult-pro > Academy-only and Youth-pro (Diff: 700 h) (NS)	Play: Adult-pro 3,956 > Youth-pro 2,689 (*)	
Machado et al. (2024a) and Machado et al. (2024b)	131 males	Childhood: Prac _futsal_ BR 486.5 > ES 88.6 (***) Prac _total_ BR 1491.2 > ES 1008 (*)	Early adolescent: Prac BR 928.3 > ES 491.6 (***) Play % BR 28.4 < ES 36.4 (*) Prac _futsal_ BR 86.2 > ES 28.1 (***) Prac _total_ BR 1005.4 > ES 519.7 (*)	
Hornig et al. (2016)	102 males	Prac per yr.: < 10 yr. 104 < 22 + yrs. 546 (*) BL (<10 yr): Play 186 > Prac 87 (*), Play 157 h > Prac 132 (NS)	Prac per yr.: 22 + yrs. 546 (*)	*Cum* hrs (1^st^ debut) NT 4532 > BL 4264 (NS)
Andrew et al. (2024)	56 females	Pro 1,557 > EA 713 (*) Practice: Pro 736 > EA 266 (*) Play: Pro 959 > EA 284 (*)	Early adolescent: Prac Pro 559 > EA 296 (*) Later adolescent: Prac Pro 795 h > EA 456 (*)	Practice and Play > Competition (*)
Ford and Williams (2012)	32 youth males	Prac: Pro 200.3 > Non 130.2 (hrs/y) (*) Play: Pro 210.2 > Non 131.6 (hrs/y) (*)		*Cum* hrs (until 15 yrs): Pro 4,840 > Non Pro 3,581 (*)
Ford et al. (2012)	328 males	Comp 37.1 h < Prac 185.7, Play 186.0 (*) Country (Prac): Mexico, Sweden > Brazil, France (*)	Prac 411.9 > Play 159.7 > Comp 66.9 (*) Country (Prac): Mexico > Brazil, England, Ghana, Sweden (*) England < Brazil, France, Portugal (*). Country (Play): Portugal > all; England> Brazil, France (*)	*Cum* hrs (until 16 yrs): All 4,553 h, Mexico > others (except Ghana, Sweden) (*) France < Mexico, Sweden (*)
Ford et al. (2009)	33 males	Prac: Pro 212, Ex 259 (NS); Elite-combined 235 > Rec 87 (*) Play: Pro 338 > Ex 148 (*); Elite-combined 243 > Rec 158(NS) Comp: Pro 40 < Ex 44 > Rec 29 (NS)		
Machado et al. (2024b)	77 females	Prac: High skill 551 > Low skill 322 (NS) Play: High skill 994 < Low skill 1909 (NS) Prac in fustal: High skill 559 < Low skill 357 h (NS) Total Prac: High skill 1,087 < Low skill 679 (NS)	Early adolescent (13–15 y): Prac in fustal: High skill 488 > Low skill 237 (*) Total prac: High skill 1,108 < Low skill 659 (*) Later adolescent (16-18y):	
Machado et al. (2020)	149 males			Per yr.: Comp High offense skill 215 > Low offense skill 108 (***) Team Prac: High offense skill 1,146 > Low offense skill 589 (***) Team Prac: High defense skill 991 > Low defense skill 481 (***) Individual Prac: High defense skill 724 > Low defense skill 473 (*)
Haugaasen et al. (2014)	745 males	*Cum* hrs: Childhood (6–8 yrs): Play: Pro > Non Pro (*) Coach-led Prac: Pro > Non Pro (*) Pro vs. Non-Pro (hours in individual, peer, match) (NS)		
O’Connor et al. (2016)	127 males			Training hrs per month: Match: Selected 5.75 < Non-Selected 6.89 (NS) Coach-led: Selected 19.01 > Non-Selected 18.66 (NS) Individual: Selected 12.41 < Non-Selected 12.45 (NS) Peer-play: Selected 17.38 < Non-Selected 19.24 (NS) Indirect: Selected 36.46 < Non-Selected 39.26 (NS)
Berry et al. (2008)	32 males			*Cum* hrs before Pro: Expert 4,185 > Less skill 3,223 (*) AFL structure: Expert 2,510 > Less-skill 2025 (NS) Deliberate Play cum hrs: Expert 2,210 > Less-skill 1,124 (NS) Deliberate Play hrs in childhood: Expert 1,039 > Less-skill 328 (*)
Andrew et al. (2022)	25 males	Skilled 1903 > Less-skilled 1,430 (NS) Individual Prac: Skilled 701.8 > Less-skilled 449 (*)	Skilled 2,532 > Less-skilled 1,690 (*) Individual Prac: Skilled 1,392 > Less-skilled649 (*)	
Charbonnet et al. (2024)	84 females	*Cum* hrs in childhood: C1 1,578 < C2 2,838 > C3 2,476 (*) Prac hrs: C1 144 < C2 598 < C3 754 (*)		*Cum* hrs in career: C1 3,866 < C2 5,419 < C3 5,614 (*) Prac hrs: C1 1,240 < C2 2,572 < C3 3,512 (*)
Zibung and Conzelmann (2013)	346 males	*Cum* hrs in Prac ≤ 12 yrs.: 967 ± 287 C1: 972 h C2:702h C3:919h C4: 1312 C5: 1212 *Cum* hrs in Play ≤ 12 yrs.: 2536 ± 1,277 C1:2057, C2:2223, C3:3175 C4: 1364, C5:4440		
Coutinho et al. (2023)	546 males	Training hrs per week (at 10-11 yrs): Football 4.12, 4.26 < Water Polo 5.27, 6.25 (*) Training hrs per week (at 12 yrs): Football 4.37 < Basketball 5.42, Volleyball 5.53, WaterPolo 6.98 (*)		
Rumpf et al. (2014)	1,075 players: 816 males, 259 females	Total hrs per week: Male: High-level 6.67–6.85 > Low-level 5.54–5.91 (U15-U19, *) U15 High-level > Low level in Technical/Tactical, Strength Training (*) U17 High-level > Low-level in Sprint (*) U19 High-level > Low-level in Technical/Tactical, strength, sprint and cool-down training (*) Across-age (High-level): Technical/Tactical U19 > U17(*) Cool down U19 > U15(*)		Female: Total hrs per week: U15 < U17 < U19 (*) U19: High-level > Low level in Endurance, Warm-up, Total (*) U17: High-level > Low level in Total hrs, Years (*)
Leite et al. (2009)	112 males	*Cum* hrs per week: 6–10 yrs.: 1–2 (2.4%) 2–3 (11.9%) 3–4(9.5%) 4–5(26.2%) ≥ 5(40.5%)	11–14 yrs.:1–2 (2.4%) 3–4(7.1%) 4–5 h (16.7%) ≥ 5(73.8%) 15-18 yrs.: 3–4 (4.8%) ≥ 5(95.2%) ≥19 yrs.:3–4 (2.4%) ≥ 5(97.6%)	
Toering et al. (2009)	444 youth males	Engagement hrs per week: Training: Elite 7.6 yrs. > Non elite 2.9 yrs. (*) Match: Elite 1.3 > 1.0(*) Years of competitive football experience: Elite:8.4 > Non-elite 8.2		
Huijgen (2013)	131 males		*Cum* Prac hrs per week: 14 yrs.: Pro 6.7 > AM 5.8 15 yrs.: Pro 6.8 > AM 6.3 16 yrs.: Pro 7.7 > AM 6.8 17 yrs.: Pro 7.4 > AM 7.0 18 yrs.: Pro 9.3 > AM 7.7	*Cum* yrs. (14–18 yrs): Pro 7.6–11.7 > AM 7.5–11.7
Gissis et al. (2006)	54 youth males			*Cum* hrs per week in Training: Elite 4.3 h > Sub elite 3.2 h > Recreational 2.7 h (*) *Cum* yrs.: Elite 5 yrs. > Sub elite 3.6 yrs. > Recreational 2.7 yrs. (*)
Helsen et al. (1998)	73 males	Individual Prac per week: International 5.2 > Provincial 3.1 Team Prac per week: International > National > Provincial (*) *Cum* hrs in Prac (13 yrs. into career): International 6,328 > National 5,220 > Provincial 4,081 h (*)		
Ford and Williams (2008)	20 males			*Cum* hrs from 6 yr-Pro: 4644.8 *Cum* hrs per year: Football-No Gaelic: 404.5 < Football-Gaelic 441.8 (NS)
Ward et al. (2007)	203 males			*Cum* hrs per year: Team Prac: Elite > Sub-elite, gap↑with age (*) Individual Prac: Elite > Sub-elite, esp. ≥U12 (*) Playful: Sub-elite > Elite (U9–U13), ≈ later (NS) Match-play: Elite > Sub-elite, all ages (*)
Koslowsky and Botelho (2010)	40 males	*Cum* prac hrs (≤12 yrs): Portugal 1,167 > Brazil 698(*)	*Cum* prac hrs (≤15 yrs): Portugal 1925 > Brazil 1,356 (*)	*Cum* prac hrs (≤18 yrs): Portugal 2,975>Brazil 2,535(*)
Hendry et al. (2014)	148 males	*Cum* hrs from (5–12 yrs): Play 1867 > Prac 1,453 (*) U13(≤12 yrs): Play 1,579–1966, Prac 1,408–1791 U15(≤12 yrs): Play 1,699 > Prac 1,357 U17(≤12 yrs): Play 1961 > Prac 1,236		
Sieghartsleitner et al. (2018)	294 males	Overall cum hrs (≤12 yrs): Play 2058 > Prac 1,128 Key clusters: enthusiasts ↑ play; club players ↑ practice		
Güllich et al. (2017)	44 males	*Cum* hrs in Prac (≤10 yrs): Strong 686 < Weak 934 (*) *Cum* hrs in Play (≤10 yrs): Strong 1,358 > Weak 881 (*)		
Güllich et al. (2020)	100 males	*Cum* hrs (T1): Prac 707 ± 268 Comp: 861 ± 760 *Cum* hrs (T1-T2): Prac 168 ± 65 Comp: 136 ± 105		

**Table 4:** Structural composition and characteristics of football-specific activities.

Author	Sample	Structure Comparison	Structural Features
Güllich (2019)	29 females	15–18y: NT Play 42% > BL 30% (**) NT Prac 23% < BL 32% (*) 19–21y: NT Play 41% > BL 31% (**) 22–25y: NT Play 41% > BL 32% (**)	Overall: Play: 30–48% Drill: 32–40% Physical 12–32%. 11–14 yrs.: NT Physical 15% < BL 25% (*) 19–21y: NT Physical 25% > NT 32% (**) 22–25y: NT Physical 27% > BL 32% (*)
Hendry and Hodges (2018)	128 males	Play (%): Adult-pro 59.26% > Youth-pro 48.56% (*)	
Machado et al. (2024a)	131 males	Play% (Early Adol): Brazil 28.4% < Spain 36.4% (*)	Practice Structure: Group tactics Brazil 26.8% > Spain 23.8% (*)
Hornig et al. (2016)	102 males	Play% (U11-22+): Prep drops 50% → 32% (**) Season drops 53% → 42% (**)	Prep Season: Play 32–50%, Skill 31–40%, Phys 13–37% Phys% (U11-22+): Rises 13% → 37% (**)
Machado et al. (2024b)	77 females	Play %: High 53.9% < Low 79.9% (Ch, *) High 38.6% < Low 59.2% (Early Adol, *) High 22.3% < Low 37.9% (Late Adol, *)	
Machado et al. (2020)	149 males		High-offensive > Low-offensive in Individual, drills, group, collective tactics (*) High-offensive > Low-offensive in low/high decision-making opportunities. (*)
Koslowsky and Botelho (2010)	40 males	Football specific domain %: Portugal 77.4% > Brazil 61.6%	

Cum. hrs = cumulative hours; h/wk = hours per week; h/yr = hours per year; Ch = childhood; Adol = adolescence; Prac = practice; Comp = competition; IndPrac = individual practice; TeamPrac = team practice; Phys = physical conditioning; Prep = preparation period; Tech/Tac = technical/tactical training.

The deliberate practice theory posits that individuals require approximately 10,000 h or ten years of deliberate practice to attain expert proficiency (Ericsson et al., 1993). However, within the football domain, owing to the distinctive nature of professional careers and temporal constraints on peak performance, athletes accumulated training hours typically fall substantially below this theoretical benchmark. Research delineates football players’ training durations framed by temporal milestones. For instance, prior inquiries indicate that male footballers generally accrue 4,500–6,000 h prior to attaining professional status (Berry et al., 2008; Ford et al., 2012; Ford and Williams, 2012; Helsen et al., 1998; Hornig et al., 2016). Conversely, studies on female football participation exhibit considerable cross-national variances; for example, Hendry et al. (2019) documented that Canadian national-level female players amassed 8,000 h of football engagement by age 19, whereas Brazilian and English counterparts registered fewer than 4,000 h. A consistent pattern across both male and female cohorts is the markedly higher training durations during adolescence compared to childhood (Table 5). Hendry and Hodges (2018)examining 128 male football players reported approximately 4,000 h in adolescence, significantly exceeding the 2,000 h in childhood. Machado et al. (2024b) corroborated this in Brazilian females, with adolescent hours approximating 2,400 versus 1,087 in childhood.

**Table 5:** Subjective ratings and perceptual-cognitive differences across training activities.

Author	Sample	Main Results	Key Findings
Hendry et al. (2019)	45 females	Challenge Rating: Na > Va, Play _Difference_ = 1.14 (**) Adol > Ch in Prac _Difference_ = 0.42 (*p* < 0.001) and Comp _Difference_ = 0.21 (***) Hrs in Challenge activities: Na > Va in Prac _Difference_ = 874.2 h and Play _Difference_ = 280.4 h (**) Adol > Ch in Prac _Difference_ = 1307.2 h, Play _Diff_ = 110.5 h, Comp _Difference_ = 189.9 h (***)	National players reported higher challenge and accumulated more moderate-to-high challenge activity than varsity players.
Andrew et al. (2024)	56 females	Child: Physical Eff: Prac (2.4/2.8) and Comp (2.5/3.1) > Play (2.2/2.3) (**). Cognitive Eff: Prac (2.9/3.0) and Comp (2.9/3.1) > Play (2.5/2.6) (**). Early Adol: Physical Eff Prac 3.0 → 3.7, Comp 3.0 → 4.0(Pro < EA)(*) Prac and Comp > Play 2.3 → 2.4 (**). Later Adol: Motivation: Comp > Play and Prac: Pro 4.0 > 3.5 and 3.8, EA 4.4 > 3.7 and 3.7 (**);	Practice and competition were rated as more physically and cognitively demanding than play, with some effort differences favoring ex-academy players during adolescence.
Hendry et al. (2018)	102 males	Coach Rating 3.4 < Player Rating 3.7 Ability Rating: Tactical < Technical, Physical (*) Academy only < Professional (Tactical, Technical, Physical Rating) (*) Youth-Pro > Adult-Pro (Creative Rating) (*) Correlations (Practice - Skill): YPro (Child): Prac–Tech (*), Prac–Creative (*) APro (Child): Prac-Phys Technical (NS) Whole Pro (career): Prac-Technical Tactical Creative (*) Correlations (Play–Skill): Child Play: No sig. Relations (NS) Youth Pro (Play%): Negative with Tactical Technical (*) Adult Pro (career Play): Positive with Tactical and Physical (NS)	Professional pathway players showed higher ability ratings and stronger practice–skill associations than academy-only players.
Machado et al. (2020)	149 males	Assessment of decision-making opportunities: Low decision making opportunities: High offense skill 271 > Low offense skill 135 (*) High decision making opportunities: High offense skill 320 > Low defense skill 140 (**) Perceptive opportunities to make decisions: High offense skill 15.6 > Low offense skill 14.1 (*) Low decision making opportunities: High defense skill 236 > Low defense skill 128 (*) High decision making opportunities: High defense skill 249 > Low defense skill 144 (**) Perceptive opportunities to make decisions: High defense skill 15.5 > Low defense skill 14.5 (*)	Higher-skilled players accumulated more practice opportunities involving decision-making demands.
Haugaasen et al. (2014)	745 males	Contribution: Deliberate > Play (ES ≈ 0.97) Pro > Non-pro (*) Relevance: Deliberate> Play (*), Pro > Non-pro (*) Enjoyment: Play > Delib (ES ≈ 1.0) Non-pro > Pro (Play), Pro > Non-pro (Deliberate) (*) Concentration: Deliberate > Play (ES ≈ 0.99) Pro≈ Non-pro (NS)	Deliberate practice was perceived as more relevant and effortful, whereas play was rated as more enjoyable.
O’Connor et al. (2016)	127 males	Perceptual–cognitive performance: % (success rate) Decision-making (%): Selected 69% > Non-Selected 59% (*) Anticipation%: Selected 63% > Non-Selected 59% (NS) Situational probability %: Selected 68% > Non-Selected 66% (NS) Pattern recognition (%): Selected 71% < Non-Selected 72% (NS) Perceptual–cognitive performance (%): Selected 65% > Non-Selected 62% (*)	Selected players showed superior decision-making accuracy and overall perceptual-cognitive performance.
Rumpf et al. (2014)	1,075 players (259 females, 816 males)	Males: High-level > Low-level in Motivation during training and game; significant across all age groups (*). Females: Only U17 game motivation: High-level > Low-level (*); other comparisons mostly NS.	Higher-level players generally reported greater motivation, especially among male players.
Toering et al. (2009)	444 males	Training perceptual: Reflection: Elite 4.09 vs. Non-elite 3.69 (*) Eff: Elite 3.05 vs. Non-elite 2.62 (*)	Elite players scored higher in reflection and effort than non-elite players.
Helsen et al. (1998)	73 males	High Relevance: Coach alone 8.28 Technical skill: 8.0 Tactics: 8.18 High Eff: Running 7.30 Weights 6.83 High enjoyment: Tactics 9.2 Technical Skills: 8.29 High Concentration: Coach alone 7.4 Tactics 7.74 Technical skill 8.42 Coaching 8.08	Technical, tactical, and coach-led activities were rated highly for relevance, enjoyment, and concentration.
Vaeyens et al. (2007a)	87 males	Training Perception/ Decision Skill: Decision Time: Elite/Sub-elite < Regional < Non-players (*) RA: Elite/Sub-elite > Regional > Non-players (*) FixOrd: Elite > Others (*) Effect of Task Constraint: 2v1 and 3v1 → faster Decision Time + fewer fixations (longer fixation duration) 3v2 and 4v3 → slower Decision Time + more fixations (shorter fixation duration) 5v3 → higher Response Accuracy, lower Decision Time (DT) (NS vs. 3v2/4v3)	Higher-level players showed faster and more accurate decisions, with task constraints shaping visual-search behavior.
Ward et al. (2007)	203 males	Enjoyment: Tech skills, Tactics, Coaching, Match-play = consistently high across ages (NS). Eff/Dedication: Elite > Sub-elite in Time/Eff (9.58 ± 0.62 vs. 7.72 ± 2.48) and Dedication (9.13 ± 1.99 vs. 6.58 ± 2.98) (*) Concentration/Relevance: Soccer-specific (Tech, Tactics, Coaching, Match) > Overall Mean (*) in Enjoyment, Eff, Concentration. Motivation Shift: Younger = Process-based enjoyment; Older = Outcome-based enjoyment (*)	Football-specific activities were rated highly, and elite players reported greater effort and dedication.
Smeeton et al. (2004)	36 males	Skilled > Less-skilled in Accuracy (*) Unstructured < Structured in Reaction time and Accuracy (*); 4-way interaction in RT (*)	Skilled players showed greater accuracy and performed better in unstructured decision-making tasks.
Hendry et al. (2014)	148 males	Motivation: Overall: Autonomous motivation and Self-determination index > Controlled motivation (*) Group diff: U17 < U13/U15 in Autonomous motivation (*) U17↓Integrated regulation and Identified regulation (*) Predictors: Play/Prac hrs ↔ Self-determination index (NS); Total Prac hrs ↔ Integrated regulation R = 0.18 (*) Academy yrs. (U17): Self-determination index ↓ (*) + Controlled motivation↑(*)	Autonomous motivation was generally high, but U17 players showed reduced autonomy and greater controlled motivation.
Vaeyens et al. (2007b)	42 males	Decision Accuracy: Skilled group 67% > Less-skilled group 52% (*) Decision Time: Skilled group 850 ms < Less-skilled group 1,050 ms (*) Visual Search Behaviors Skilled group → fewer but longer fixations (3.5 fixations, 320 ms each) Less-skilled group → more but shorter fixations (5.5 fixations, 190 ms each) Skilled players alternate fixation more effectively between ball, space, and teammates (*)	Skilled players made faster, more accurate decisions and used more efficient visual-search strategies.

Eff = Effort; ↑ = significantly higher; ↓ = significantly lower; ↔ = mutual influence.

Across different activity types, studies on both male and female football players have consistently demonstrated that the hours of coach-led practice and play is significantly higher than that of competitions (Andrew et al., 2024; Hendry and Hodges, 2018; Roca et al., 2012; Zibung and Conzelmann, 2013). The proportion of coach-led practice and play shifts across developmental stages. The duration of play exceeds that of coach-led practice during childhood (Hendry and Hodges, 2018; Roca et al., 2012); However, from adolescence onwards into adulthood, the proportion of play gradually declines and becomes lower than that of coach-led practice (Ford et al., 2012; Güllich, 2019; Hendry et al., 2019; Machado et al., 2024b). For example, Ford et al. (2012) investigated 7 countries and found that the threshold for the transition from play to practice hours occurs around ages 9 to 11. Before age 9, the activity of play predominates, while after age 11, practice hours increase.

Differences in accumulated hours among athletes of varying skill levels have long been a central focus for scholars investigating the diversification of athletic career paths. Most studies uphold the notion that higher-level athletes dedicate more time in soccer-specific activities (Hornig et al., 2016; Roca et al., 2012), particularly in the hours of coach-led practice and play (Andrew et al., 2024; Ford and Williams, 2012; Gissis et al., 2006; Helsen et al., 1998; Hendry et al., 2019; Hendry and Hodges, 2018; Huijgen, 2013). However, other studies have found that the accumulated hours of participation in specific sports and stages among higher-level athletes is not always significantly higher than that of lower-level athletes (Ford et al., 2009; Ford and Williams, 2012; Güllich, 2019; Haugaasen et al., 2014). For instance, Güllich (2019) found that national-level athletes spent less practice during childhood than professional-level athletes. Ford and Williams (2012)observed no significant differences in practice, play or competition hours between professional and non-professional athletes in adulthood. Haugaasen et al. (2014) reported no significant differences between professional and non-professional athletes in individual practice, peer training, or competition duration. It should be noted that discrepancies in findings often stem from methodological choices (e.g., retrospective questionnaires versus multilevel models), sample characteristics (gender, developmental stage, and skill level stratification), and variations in training systems across national and cultural contexts. These factors collectively contribute to inconsistent findings regarding specialized training duration between elite and recreational athletes.

The composition of football-specific activities further reveals how training emphasis changes across development. Research on German cohorts reveals a progressive decline in playful training proportions concomitant with ascending physical conditioning ratios as age increases (Güllich, 2019; Hornig et al., 2016). Machado et al. (2024a) reported that Brazilian players devoted more time to individual practice, pairs practice, drills, group tactics, collective tactics, and training with high decision-making demands than Spanish players, with particularly large differences for decision-rich training. Furthermore, some studies have emphasized the importance of futsal in the development of talent, providing varied early motor experiences. In confined spaces, at high speeds and with increased demands on decision-making, futsal has been shown to significantly enhance perceptual-motor skills, passing proficiency and tactical acumen. This “donor sport” attribute renders futsal an indispensable adjunct in football talent development frameworks, pivotal for elite attainment (Machado et al., 2020; Machado et al., 2024b; Oppici et al., 2019; Travassos et al., 2018; Yiannaki et al., 2018).

Additionally, research on female football has identified a unique developmental phenomenon: elite female football players often possess a distinct type of training experience involving mixed-gender training activities. Andrew et al. (2024)found that over half of professional English female football players participated in mixed-gender training during childhood. Güllich (2019) also highlighted the significance of mixed-gender participation, noting that national-level female footballers initiated cross-gender training at an earlier age and accumulated more hours of such training than professional-level counterparts. This phenomenon may be attributed to the underdeveloped state of early female football training systems, whereby many female athletes compensated for insufficient training resources during childhood by engaging in play or training with boys.

### 4.3 Subjective ratings and perceptual differences

Research on subjective ratings of football-specific activities typically uses validated scales to quantify athletes’ perceptions across activity types; these indices capture perceived training quality and experiential states and are conceptually linked to deliberate practice (Ericsson et al., 1993). In general, coach-led practice and competition attract higher ratings for challenge/effort than play (Toering et al., 2009; Ward et al., 2007), whereas play is consistently associated with greater enjoyment (Andrew et al., 2024; Haugaasen et al., 2014; Hendry et al., 2019). Andrew et al. (2024) reported significantly higher physical and cognitive effort during competition and practice than play, particularly in early adolescence. Taken together, these findings suggest that structured, feedback-rich contexts are perceived as more demanding and task-relevant, thereby fostering motivation and attentional focus. By contrast, although play activities predominate during childhood, their lower challenge ratings may constrain skill optimization, potentially attributable to the absence of formal guidance (Hendry et al., 2019).

Between-level comparisons, higher-level athletes generally exhibit superior subjective ratings compared to the lower-level players, particularly in dimensions such as challenge (Hendry et al., 2019), effort (Andrew et al., 2024), motivation (Hendry et al., 2014; Rumpf et al., 2014; Ward et al., 2007), and reflection (Toering et al., 2009). This pattern implies that elite players are more inclined to view training as high-intensity and highly relevant endeavors, fostering sustained engagement and skill enhancement. Hendry et al. (2019)demonstrated that national-team players achieved higher challenge ratings and accumulated more hours in medium-to-high challenge coach-led practice. However, not all differences are consistent, Andrew et al. (2024) observed that former academy players displayed higher physical effort ratings during adolescence, possibly as they exerted additional effort to compensate for resource limitations.

Disparities in perceptual performance across different levels of ability are predominantly evident in skills such as decision-making and pattern recognition. These skills serve as objective indicators of the efficacy of training programs (Helsen et al., 1998; Hendry et al., 2018; Machado et al., 2020; O’Connor et al., 2016; Smeeton et al., 2004; Vaeyens et al., 2007a; Vaeyens et al., 2007b). Empirical data reveal that elite players demonstrate superior decision-making skills during training; for example, Vaeyens et al. (2007b) found that the elite athletes had greater decision accuracy and made decisions more quickly in video-based tests. Machado et al. (2020) also revealed that, among youth players, those with high offensive skills achieved higher performance ratings in situations requiring quick decisions, outperforming those with lower skills in tactical decision-making. Other studies corroborate this trend, showing that higher-level players display enhanced decision accuracy and pattern recognition abilities (Smeeton et al., 2004), particularly in dynamic, complex and unstructured scenarios (O’Connor et al., 2016). However, variations in perceptual performance between levels are modulated by factors such as task complexity (Vaeyens et al., 2007a) methodological approaches (O’Connor et al., 2016) and training cultural contexts (Machado et al., 2020). Furthermore, most samples are male dominated, with limited female data (Hendry et al., 2019), which may restrict the generalisability of findings.

### 4.4 Multi-Sport participation

In the early stages of soccer talent development, whether to focus on specialized training or expose players to a variety of sports has long been a central debate in coaching philosophy. Sport diversity typically refers to participating in multiple sports during childhood and adolescence rather than prematurely specializing in a single discipline (Ford et al., 2009). This approach is widely recognized for facilitating skill transfer, reducing the risk of overtraining and injury, extending athletic careers, and promoting overall physical literacy (Güllich, 2019).

Existing research indicates that most athletes have participated in multiple sports, particularly during childhood (Table 6). The age of initiation into other sports generally occurs later than that for soccer, typically ranging from 7 to 10 years old (Andrew et al., 2024; Ford et al., 2009; Haugaasen et al., 2014; Hornig et al., 2016). The termination of this diversification typically occurs between ages 13 and 16(Andrew et al., 2024; Güllich, 2019; Haugaasen et al., 2014; Ward et al., 2007). For instance, Haugaasen et al. (2014) surveyed 745 soccer players and found that elite soccer players began specialization at 9.3 years old and completed it by 13.1 years old. Similarly, Ward et al. (2007) found that U17–U18 players terminated multi-sport involvement at approximately 16 years.

**Table 6:** Multi-sport participation and cross-sport engagement patterns of football players.

Author	Sample	Main Results	Key Findings
Roca et al. (2012)	64 males	Start age: High 11.5; Low 10.8; Rec 11.1 (NS) No.of sports: Ch: High 2.1, Low 2.2, Rec 2.2 (NS) Adol: High 2.7, Low 2.4, Rec 2.6 (NS)	No significant differences were found in other-sport initiation age or number of other sports across groups.
Hendry et al. (2019)	45 females	No. of sports: NT < Var. Diff = 2 (**) Ch: All: 4 ± 2.5, Nat: 3 ± 2.1, Var:5 ± 2.4 (NS) Ado: All: 4 ± 2.5, Nat: 4 ± 2.6, Var:5 ± 2.3 (NS) *Cum* hrs: Football practice > Other sports, Diff = 3238.5 (***)	NT players participated in fewer other sports, while football practice hours exceeded other-sport involvement.
Güllich (2019)	29 females	Start age: NT 6.8 ± 3.2, BL 7.4 ± 2.5 (NS) End age: 15.9 ± 5.7, BL 14.1 ± 3.0(NS) Other sports duration: NT 7.2 ± 6.6 yrs., BL 6.0 ± 4.1 yrs. (NS) other sports practice: NT 5.4 ± 5.3 yrs. > BL 1.5 ± 2.8 yrs. (*) Exclusive specialization age: NT 14.1 ± 7.2 vs. BL 10.6 ± 5.5 (NS) Multi-sport span: NT 9.7 ± 7.2 yrs. > BL 5.1 ± 5.5 yrs. (*)	NT players accumulated more other-sport practice and maintained a longer multi-sport span than BL players.
Hendry and Hodges (2018)	128 males	No. of sports: 4–5 (NS) Hrs in soccer activities (5-8 yrs): 2.88–4.63 h/wk. > Other sports 0.97–1.25 h/wk. (***) Hrs in soccer activities (9–12 yrs): 6.38–9.23 h/wk. > Other sports 3.06–3.83 h/wk. (***) Specialization: <10% early-specialized; none in Adult-pro, few in Youth-pro-only.	Other-sport involvement was similar across pathways, while football-specific activity was consistently greater than other-sport participation.
Machado et al. (2024a)	131 males	No. of sports: Childhood Brazil 1.3 > Spain 0.9 (**) Early adolescent Brazil 0.8 > Spain 0.4 (***)	Brazilian players participated in more other sports than Spanish players during childhood and early adolescence.
Hornig et al. (2016)	102 males	Start age: NT + 1stBL:7.8 ± 2.6 NT: 8.6 ± 2.1 1stBL:7.2 ± 2.8 a.m.: 9.7 ± 5.4 (NS) Start Prac: NT + 1stBL:8.2 ± 2.0 NT: 8.7 ± 1.7 1stBL:7.8 ± 2.3 a.m.: 9.4 ± 4.4 (NS) No. of sports: NS Adolescent: Prac NT > 1^st^ BL, AM (*)	Other-sport initiation did not differ across groups, but NT players accumulated more adolescent other-sport practice.
Andrew et al. (2024)	56 females	Start age: Pro 9.6 < EA 10.9 (NS) End age: Pro 13.9 < EA 15.4 (NS) No. of sports: Pro: 1.2–1.4 EA: 1.6–1.7 (NS) Childhood: Pro prac, play > EA Early adolescent Comp hrs Pro < EA Later adolescent Prac, Comp hrs Pro < EA	Pro and EA players showed similar other-sport timing and number, but differed in stage-specific practice, play, and competition hours.
Ford and Williams (2012)	32 males	No. of sports in Childhood: Pro 4.3 < Non Pro 4.6 (NS) Early adolescent Pro 4.4 < Non Pro 3.8 (NS) *Cum* hrs in other sports by 15: Pro 992 vs. Non 879 (NS)	No significant differences were found in other-sport number or accumulated other-sport hours by age 15.
Ford et al. (2012)	328 males	No. of other sports: Childhood: All 2.32 ± 1.63; England 4.40 > all others; Sweden > Brazil (*). Adolescent: All 2.52 ± 1.76; England > others (except France) (*)	English players reported the broadest other-sport participation, whereas Brazilian players showed greater futsal involvement.
Ford et al. (2009)	33 males	Start age: All: 9.3 ± 2.2 (No group diff) No. of other sports: All 1.5 ± 1.3 (No group diff) *Cum* hrs: Elite-combined 183 < Rec 403 (NS) Pro 161 < Ex 204 (NS)	Other-sport initiation, number of sports, and accumulated hours did not differ significantly across groups.
Güllich et al. (2020)	100 males	Youth-led play→football skill learning progress (NS) Coach-led prac →football skill learning progress (*) Comp →football skill learning progress (*) *Cum* football hrs → football skill learning progress (NS) Other sports: Coach-led prac →football skill learning progress (Years before T1, Hours before T1, T1-T2) (**) Comp→football skill learning progress (Years before T1) (**)	Football skill development was more strongly associated with coach-led practice in other sports than with cumulative football hours.
Machado et al. (2024b)	77 females	No. of sports: Childhood: High skill 1.1 > Low skill 0.8 (NS) Early adolescent: High skill 1.2 < Low skill 1.4 (NS) Later adolescent: High skill 0.8 < Low skill 1.2 (NS)	Decision-making skill was not differentiated by the number of other sports, but by a specialized football–futsal pathway.
Haugaasen et al. (2014)	745 males	Start age: Pro 9.3 ± 2.4 < Non-pro 8.8 ± 2.2(NS) End age: Pro 13.1 ± 1.9 > Non-pro 12.1 ± 2.2 (*) No. of sports: Pro 1.1 > Non-Pro 1.0 (NS) *Cum* hrs in other sports: Pro ≈ Non-pro (NS) *Cum* football hrs > other sports across 6–21 y (*) *Cum* football hrs (10y, 13y): Multi-sport > Football-only (*) *Cum* football hrs (20y): Football-only > Multi-sport (*) Training Rating: Team-sim > Team-dis: Relev Concent; Enjoy (*) Team-sim > Indiv: Relev, Enjoy (*) Indiv > Team-dis: Relev, Enjoy (*) Pro ≈ Non-pro: (NS)	Other-sport hours did not differ by professional status, while team-similar sports received higher experiential ratings.
Taylor et al. (2024)	60 males	About half of English academies implemented multi-sports training, ~1–2 sessions per week (~40 min). Activities included tag games, handball, and gymnastics. Practitioners perceived benefits for fundamental movement skills, coordination, agility, and psychosocial skills; players enjoyed the sessions. Barriers included lack of time, resources, and institutional support.	Multi-sport training was valued for broad developmental benefits, but implementation was constrained by time, resources, and institutional support.
O’Connor et al. (2016)	127 males	No. of other sports: Selected 3.4 < Non-Selected 4.0 (NS)	Selected and non-selected players did not differ significantly in the number of other sports played.
Berry et al. (2008)	32 males	No. of other sports: Expert 3.6 > Less-skilled 2.5 (*) Non invasion structure. Hrs: Expert 1,359 h > Less-skilled 445 h (*)	Expert players engaged in more invasion sports and accumulated greater non-AFL invasion-sport experience.
Andrew et al. (2022)	25 males	No. of other sports: Childhood Skilled 1.6 < Less- skilled 2.0 (NS) Early adolescent Skilled 2.1 > Less-skilled 1.9 (NS) *Cum* hrs in Childhood Skilled 692 h < Less-skilled 722 (NS) *Cum* hrs in Early adolescent: Skilled 540 h < Less-skilled 703 (NS)	Other-sport number and accumulated hours did not differ significantly between skilled and less-skilled players.
Charbonnet et al. (2024)	84 females	*Cum* hrs in Childhood (C1 384 h) < C2 603h < C3 438h (NS) *Cum* hrs in career (C1 898 h) > C2 768h > C3 559 h (NS)	Other-sport hours did not differ significantly across female player cohorts.
Zibung and Conzelmann (2013)	346 males	Index of other sports engagement (0–3): Overall:0.81 ± 1.06 C1: 1.27 ± 0.45 C2: 1.49 ± 0.83 C3: 3.62 ± 0.5 C4:2.58 ± 0.9 C5:1.22 ± 0.52	Both diversified and specialized developmental pathways were observed among elite players.
Coutinho et al. (2023)	546 males	No. of other sports engagement:1.18 ± 0.53 Type of sports: football players were more involved in team games and less in coordination and gymnastic sports	Football players were more involved in team games and less involved in coordination or gymnastic sports.
Leite et al. (2009)	112 males	No. of other sports engagement: 6–10 yrs.: n = 1 (33.3%) n = 2 (2.4%) 11–14 yrs.: n = 1 (9.5%) 15-18 yrs.: n = 1 (2.4%) Type of sports prac: Main team sport: 6–10 yrs.: 54.7% 11–14 yrs.: 88.1% 15-18 yrs.:97.6% ≥19 yrs.:100% Combination of several sports: 6–10 yrs.: 35.7% 11–14 yrs.: 14.4% 15-18 yrs.:2.4%	Other-sport participation was limited and mainly involved team sports.
Ford and Williams (2008)	20 males	Start age: Football-No Gaelic 10 ± 2.6 < Football-Gaelic 8.5 ± 1.2 (NS) End age: Football-No Gaelic 13.8 ± 2.0 < Football-Gaelic 14.0 ± 1.6 (NS) No. of other sports (6 yrs-Pro): Football-No Gaelic 2.5 ± 1.7 < Football-Gaelic 4 ± 1.7 (NS) No. of other sports (across 3 stages): Football-No Gaelic 1.5 ± 1.1 < Football-Gaelic 3.1 ± 1.4 (*)	Football-Gaelic players reported broader other-sport participation across developmental stages than football-only players.
Ward et al. (2007)	203 males	No. of other sports: Elite ≈ Sub-elite (~2, NS); U17 > U15 (*) Start age: U9 (~6.5y), U10 (~7.5y) < U14 + (~9.8y) (*) (Elite ≈ Sub-elite, NS) End age: U17 ~ 16.0y, U18 ~ 16.3y; no skill diff (NS) Level in other sports: Elite 1.74 > Sub-elite 1.39 (*) but small diff (mainly rec/amateur)	Elite and sub-elite players showed similar other-sport timing and number, but elites reached slightly higher other-sport levels.
Smeeton et al. (2004)	36 males	Sport transfer: football↔Hockey (bi-directional transfer) (*) Football/Hockey → Volleyball (uni-directional) (NS)	Transfer was stronger between structurally similar invasion games than between dissimilar team sports.
Koslowsky and Botelho (2010)	40 males	*Cum* hrs in other sports: (≤12 yrs): Portugal 728 h< Brazil 1,136 h (*) *Cum* hrs in other sports: (≤15 yrs): Portugal 894 h< Brazil 1,474 h (*) *Cum* hrs in other sports: (≤18 yrs): Portugal 961 h< Brazil 1,605 h (*) Non-specific domain%: Portugal 22.6% >Brazil 38.4%	Brazilian players accumulated more other-sport hours, reflecting greater non-specific or diversified sport involvement.
Memmert and Roth (2007)	135 males	Sport transfer: Hand→Football R = 0.33 (*) Football→Hockey R = 0.42 (*)	Football-specific and diversified training showed transfer benefits for creative performance across related team sports.
Hendry et al. (2014)	148 males	No. of other sports: U12:5.4 ± 2.2 Range: 1–11 U15:5.3 ± 2.6 Range: 1–14 U18:5.4 ± 2.2 (NS) Range: 1–9	Players had broad multi-sport backgrounds, but diversification declined with age and academy involvement.
Sieghartsleitner et al. (2018)	294 males	Overall hrs in other sports: 1837 h (poly-sportive ↑diversity; abstainers ↓all)	Multi-sport participation was common, but showed limited predictive value for attaining the highest performance level.
Güllich et al. (2017)	44 males	No. of other sports: Strong 2.3 ± 2.4 > Weak 1.1 ± 1.2 (*) types of mainly invasion and net/wall games *Cum* hrs in other sports (≤10 yrs): Strong prac 219 h > Weak 41 h (*)	Strong responders showed greater youth sport diversification, especially in invasion and net/wall sports.

No. = number; Team-sim = team-similar sports; Team-dis = team-dissimilar sports; Indiv = individual sports; Relev = relevance; Concent = concentration.

Further studies reveal that elite players typically participate in 2–5 additional sports on average (Ford and Williams, 2008; Hendry et al., 2014, 2019; Hendry and Hodges, 2018; O’Connor et al., 2016). Several investigations have noted no significant differences in the number of other sports engaged during childhood compared to adolescence (Andrew et al., 2022; Ford et al., 2012; Machado et al., 2024a, 2024b). The accumulated duration of involvement in other sports is estimated at approximately 800–1,200 h (Ford and Williams, 2012; Hendry and Hodges, 2018), substantially less than football-specific engagement. Hendry and Hodges (2018) observed that time spent in other sports constitutes less than half the duration of football-specific training.

Comparative analyses of multi-sport participation across different competitive levels indicate no significant differences in the number of additional sports engaged in (Andrew et al., 2024; Ford and Williams, 2012; Hornig et al., 2016). Variations primarily manifest in participation duration and type of sports. Research suggests elite athletes typically exhibit longer participation spans and richer experiences across multiple sports (Güllich, 2019). Furthermore, Güllich et al. (2017) propose that elite athletes accumulate longer training durations in other sports. Regarding activity types, high-level athletes in childhood and early adolescence frequently engage in invasion sports (Berry et al., 2008; Güllich et al., 2017), which enhance tactical awareness; while tennis/squash-type sports help improve hand-eye coordination, reaction speed, and rhythm control, offering complementary benefits to soccer-specific perception and anticipatory movement skills (Güllich et al., 2017). In contrast, lower-level players often specialize prematurely or engage in narrower participation, which may limit their developmental potential in perceptual decision-making and creativity (Leite et al., 2009).

## 5 Limitation

Several limitations should be acknowledged. First, although this review followed PRISMA guidelines and employed a rigorous quality appraisal, its scope was restricted to peer-reviewed English-language publications indexed in three major databases. This may have excluded relevant studies published in other languages, and the heterogeneity of included studies prevented quantitative meta-analysis. Furthermore, the reliance on retrospective self-reported data in most primary studies raises concerns regarding recall bias and the accuracy of developmental timelines. More importantly, the limitations of existing literature constrain the conclusions that can be drawn. Current evidence is dominated by male, European cohorts, with female athletes and non-European populations largely underrepresented. Methodologically, most studies adopt cross-sectional or retrospective designs, with a lack of prospective longitudinal research that could establish causal relationships. Measures of training structure, perceptual ratings, and multi-sport engagement also vary considerably across studies, limiting comparability and synthesis. Collectively, these constraints highlight the need for broader, more diverse, and methodologically robust investigations to advance the understanding of football players’ developmental pathways.

## 6 Conclusion

This review demonstrates that elite football players tend to engage with sport and join professional teams at earlier ages than their lower-level counterparts. Their developmental pathways are frequently characterized by early diversification, with playful and unstructured activities dominating childhood, extensive engagement in other sports—particularly invasion-type team games—and a subsequent shift toward football-specific deliberate practice during adolescence. For female players, mixed-gender training emerged as a unique and valuable developmental feature compensating for limited resources. Besides, variations in milestone timing, training patterns, and perceptual experiences were observed across gender, competitive level, and cultural contexts. These inconsistencies can be partly attributed to methodological choices, retrospective study designs, and sample characteristics that limit the generalizability of findings. Future research should therefore prioritize longitudinal and gender-comparative designs, along with cross-cultural investigations, to more accurately and comprehensively capture the dynamics of athlete development. Such efforts will provide stronger evidence to inform the optimization of talent development systems and the formulation of effective policies in football.

## Data Availability

The original contributions presented in the study are included in the article/supplementary material, further inquiries can be directed to the corresponding author/s.

## References

[ref1] Afonso P. Forte P. Branquinho L. Ferraz R. Garrido N. D. Teixeira J. E. (2025). Shaping Training Load, Technical-Tactical Behaviour, and Well-Being in Football: A Systematic Review. Sports 13:244. doi: 10.3390/sports13080244, 40863753 PMC12390603

[ref2] Andrew M. Baptiste G. Z. Reeves M. J. Roberts S. J. McRobert A. P. Ford P. R. (2022). The developmental activities of skilled youth CONCACAF soccer players and the contribution of their development system. Int. J. Sports Sci. Coach. 17, 1363–1377. doi: 10.1177/17479541211061036

[ref3] Andrew M. Ford P. R. Alder S. E. Champ F. M. Brownlee T. E. Datson N. . (2024). Talent development in female soccer: Developmental activities of professional players in England. J. Sports Sci. 42, 853–864. doi: 10.1080/02640414.2024.2356434, 38916272

[ref4] Baxter-Jones A. D. G. Maffulli N. (2003). Parental influence on sport participation in elite young athletes. J. Sports Med. Phys. Fitness 43, 250–255, 12853909

[ref5] Berry J. Abernethy B. Côté J. (2008). The Contribution of Structured Activity and Deliberate Play to the Development of Expert Perceptual and Decision-Making Skill. J. Sport Exerc. Psychol. 30, 685–708. doi: 10.1123/jsep.30.6.685 19164836

[ref6] Burgess D. J. Naughton G. A. (2010). Talent Development in Adolescent Team Sports: A Review. Int. J. Sports Physiol. Perform. 5, 103–116. doi: 10.1123/ijspp.5.1.103, 20308701

[ref7] Charbonnet B. Schmid M. J. Örencik M. Van Niekerk E. Conzelmann A. (2024). The road to excellence in women’s football: A retrospective cohort study over the last 30 years with Swiss national players. Sci. Med. Footb. 8, 374–385. doi: 10.1080/24733938.2023.2279531, 37921193

[ref8] Christensen M. K. Sørensen J. K. (2009). Sport or school? Dreams and dilemmas for talented young Danish football players. Eur. Phys. Educ. Rev. 15, 115–133. doi: 10.1177/1356336X09105214

[ref9] Côté J. Vierimaa M. (2014). The developmental model of sport participation: 15 years after its first conceptualization. Sci Sports 29, S63–S69. doi: 10.1016/j.scispo.2014.08.133

[ref10] Coutinho P. Ramos A. Afonso J. Bessa C. Ribeiro J. Davids K. . (2023). To Sample or to Specialise? Sport Participation Patterns of Youth Team Sport Male Players. Children 10:729. doi: 10.3390/children10040729, 37189977 PMC10136443

[ref11] Downes M. J. Brennan M. L. Williams H. C. Dean R. S. (2016). Development of a critical appraisal tool to assess the quality of cross-sectional studies (AXIS). BMJ Open 6:e011458. doi: 10.1136/bmjopen-2016-011458, 27932337 PMC5168618

[ref12] Ericsson K. A. Krampe R. T. Tesch-Römer C. (1993). The role of deliberate practice in the acquisition of expert performance. Psychol. Rev. 100, 363–406. doi: 10.1037/0033-295X.100.3.363

[ref13] Ford P. R. Carling C. Garces M. Marques M. Miguel C. Farrant A. . (2012). The developmental activities of elite soccer players aged under-16 years from Brazil, England, France, Ghana, Mexico, Portugal and Sweden. J. Sports Sci. 30, 1653–1663. doi: 10.1080/02640414.2012.701762, 22788752

[ref14] Ford P. R. Ward P. Hodges N. J. Williams A. M. (2009). The role of deliberate practice and play in career progression in sport: The early engagement hypothesis. High Abil. Stud. 20, 65–75. doi: 10.1080/13598130902860721

[ref15] Ford P. R. Williams A. M. (2008). The Effect of Participation in Gaelic Football on the Development of Irish Professional Soccer Players. J. Sport Exerc. Psychol. 30, 709–722. doi: 10.1123/jsep.30.6.709, 19164837

[ref16] Ford P. R. Williams M. A. (2011). No Relative Age Effect in the Birth Dates of Award-Winning Athletes in Male Professional Team Sports. Res. Q. Exerc. Sport 82, 570–573. doi: 10.1080/02701367.2011.10599790, 21957716

[ref17] Ford P. R. Williams A. M. (2012). The developmental activities engaged in by elite youth soccer players who progressed to professional status compared to those who did not. Psychol. Sport Exerc. 13, 349–352. doi: 10.1016/j.psychsport.2011.09.004

[ref18] Gissis I. Papadopoulos C. Kalapotharakos V. I. Sotiropoulos A. Komsis G. Manolopoulos E. (2006). Strength and Speed Characteristics of Elite, Subelite, and Recreational Young Soccer Players. Res. Sports Med. 14, 205–214. doi: 10.1080/15438620600854769, 16967772

[ref19] Gledhill A. Harwood C. Forsdyke D. (2017). Psychosocial factors associated with talent development in football: A systematic review. Psychol. Sport Exerc. 31, 93–112. doi: 10.1016/j.psychsport.2017.04.002

[ref20] Güllich A. (2014). Selection, de-selection and progression in German football talent promotion. Eur. J. Sport Sci. 14, 530–537. doi: 10.1080/17461391.2013.858371 24245783

[ref21] Güllich A. (2019). “Macro-structure” of developmental participation histories and “micro-structure” of practice of German female world-class and national-class football players. J. Sports Sci. 37, 1347–1355. doi: 10.1080/02640414.2018.1558744, 30582400

[ref22] Güllich A. Cronauer R. Diehl J. Gard L. Miller C. (2020). Coach-assessed skill learning progress of youth soccer players correlates with earlier childhood practice in other sports. Int. J. Sports Sci. Coach. 15, 285–296. doi: 10.1177/1747954120912351

[ref23] Güllich A. Kovar P. Zart S. Reimann A. (2017). Sport activities differentiating match-play improvement in elite youth footballers – a 2-year longitudinal study. J. Sports Sci. 35, 207–215. doi: 10.1080/02640414.2016.1161206 27018979

[ref24] Güllich A. Macnamara B. N. Hambrick D. Z. (2022). What Makes a Champion? Early Multidisciplinary Practice, Not Early Specialization, Predicts World-Class Performance. Perspect. Psychol. Sci. 17, 6–29. doi: 10.1177/1745691620974772, 34260336

[ref25] Haugaasen M. Toering T. Jordet G. (2014). From childhood to senior professional football: A multi-level approach to elite youth football players’ engagement in football-specific activities. Psychol. Sport Exerc. 15, 336–344. doi: 10.1016/j.psychsport.2014.02.007 25357261

[ref26] Helsen W. F. Starkes J. L. Hodges N. J. (1998). Team Sports and the Theory of Deliberate Practice. J. Sport Exerc. Psychol. 20, 12–34. doi: 10.1123/jsep.20.1.12

[ref27] Hendry D. T. Crocker P. R. E. Hodges N. J. (2014). Practice and play as determinants of self-determined motivation in youth soccer players. J. Sports Sci. 32, 1091–1099. doi: 10.1080/02640414.2014.880792, 24479811

[ref28] Hendry D. T. Hodges N. J. (2018). Early majority engagement pathway best defines transitions from youth to adult elite men’s soccer in the UK: A three time-point retrospective and prospective study. Psychol. Sport Exerc. 36, 81–89. doi: 10.1016/j.psychsport.2018.01.009

[ref29] Hendry D. T. Williams A. M. Ford P. R. Hodges N. J. (2019). Developmental activities and perceptions of challenge for National and Varsity women soccer players in Canada. Psychol. Sport Exerc. 43, 210–218. doi: 10.1016/j.psychsport.2019.02.008

[ref30] Hendry D. T. Williams A. M. Hodges N. J. (2018). Coach ratings of skills and their relations to practice, play and successful transitions from youth-elite to adult-professional status in soccer. J. Sports Sci. 36, 2009–2017. doi: 10.1080/02640414.2018.1432236, 29400614

[ref31] Hornig M. Aust F. Güllich A. (2016). Practice and play in the development of German top-level professional football players. Eur. J. Sport Sci. 16, 96–105. doi: 10.1080/17461391.2014.982204, 25440296

[ref32] Huijgen B. C. H. (2013). Technical Skills, the Key to Succes? A Study on Talent Development and Selection of Youth Soccer Players

[ref33] Koslowsky M. Botelho M. D. C. (2010). Domains in the practice of the football learning: Comparative study among football athletes of junior category in Portugal and Brazil. J. Hum. Sport Exerc. 5, 400–410. doi: 10.4100/jhse.2010.53.10

[ref34] Leite N. Baker J. Sampaio J. (2009). Paths to expertise in Portuguese national team athletes. J. Sports Sci. Med. 8, 560–566, 24149598 PMC3761533

[ref35] MacDonald D. J. King J. Côté J. Abernethy B. (2009). Birthplace effects on the development of female athletic talent. J. Sci. Med. Sport 12, 234–237. doi: 10.1016/j.jsams.2007.05.015, 17889609

[ref36] Machado G. González-Víllora S. Pastor-Vicedo J. C. Teoldo I. (2024a). Mapping talent pathways: A comparative study of developmental activities and practice structure in Brazilian and Spanish U-18 elite youth male soccer players. Int. J. Sports Sci. Coach. 19, 2006–2015. doi: 10.1177/17479541241241487

[ref37] Machado G. González-Víllora S. Sarmento H. Teoldo I. (2020). Development of Tactical Decision-making Skills in Youth Soccer Players: Macro- and Microstructure of Soccer Developmental Activities as a Discriminant of Different Skill Levels. Int. J. Perform. Anal. Sport 20, 1072–1091. doi: 10.1080/24748668.2020.1829368

[ref38] Machado G. González-Víllora S. Teoldo I. (2024b). Contribution of deliberate practice, play, and futsal to the acquisition of decision-making skills in Brazilian professional female soccer players. Int. J. Sport Exerc. Psychol. 22, 756–774. doi: 10.1080/1612197X.2022.2161101 37665910

[ref39] Memmert D. Roth K. (2007). The effects of non-specific and specific concepts on tactical creativity in team ball sports. J. Sports Sci. 25, 1423–1432. doi: 10.1080/02640410601129755 17786695

[ref40] Moher D. Liberati A. Tetzlaff J. Altman D. G., & The PRISMA Group. (2009). Preferred reporting items for systematic reviews and meta-analyses: The PRISMA statement. PLoS Med., 6:e1000097. doi:doi: 10.1371/journal.pmed.1000097 19621072 PMC2707599

[ref41] Morganti G. Brustio P. R. Ruscello B. Apollaro G. Padua E. Kelly A. L. (2024). Birth Advantages in Male Italian Soccer: How They Influence Players Youth Career and Their Future Career Status. Sports 12:103. doi: 10.3390/sports12040103, 38668571 PMC11054811

[ref42] O’Connor D. Larkin P. Mark Williams A. (2016). Talent identification and selection in elite youth football: an Australian context. Eur. J. Sport Sci. 16, 837–844. doi: 10.1080/17461391.2016.1151945 26923813

[ref43] Oppici L. Panchuk D. Serpiello F. R. Farrow D. (2019). Futsal task constraints promote the development of soccer passing skill: Evidence and implications for talent development research and practice. Sci. Med. Footb. 3, 259–262. doi: 10.1080/24733938.2019.1609068

[ref44] Roca A. Williams A. M. Ford P. R. (2012). Developmental activities and the acquisition of superior anticipation and decision making in soccer players. J. Sports Sci. 30, 1643–1652. doi: 10.1080/02640414.2012.701761, 22769067

[ref45] Rumpf M. C. Schneider A. S. Schneider C. Mayer H. M. (2014). Training Profiles and Motivation of Male and Female Youth Soccer Players. Int. J. Sports Sci. Coach. 9, 207–216. doi: 10.1260/1747-9541.9.1.207

[ref46] Schroepf B. Lames M. (2018). Career patterns in German football youth national teams – A longitudinal study. Int. J. Sports Sci. Coach. 13, 405–414. doi: 10.1177/1747954117729368

[ref47] Sieghartsleitner R. Zuber C. Zibung M. Conzelmann A. (2018). “The Early Specialised Bird Catches the Worm!” – A Specialised Sampling Model in the Development of Football Talents. Front. Psychol. 9:188. doi: 10.3389/fpsyg.2018.00188, 29515500 PMC5826374

[ref48] Smeeton N. J. Ward P. Williams A. M. (2004). Do pattern recognition skills transfer across sports? A preliminary analysis. J. Sports Sci. 22, 205–213. doi: 10.1080/02640410310001641494, 14998098

[ref49] Sweeney L. Horan D. MacNamara Á. (2021). Premature Professionalisation or Early Engagement? Examining Practise in Football Player Pathways. Front. Sports Active Living 3:660167. doi: 10.3389/fspor.2021.660167, 34164620 PMC8215134

[ref50] Taylor J. M. Quigley C. Madden J. Wright M. D. (2024). Multi-sports training in English soccer academies: A survey exploring practices, practitioner perspectives, and barriers to use. Int. J. Sports Sci. Coach. 19, 1671–1679. doi: 10.1177/17479541231210746

[ref51] Toering T. T. Elferink-Gemser M. T. Jordet G. Visscher C. (2009). Self-regulation and performance level of elite and non-elite youth soccer players. J. Sports Sci. 27, 1509–1517. doi: 10.1080/02640410903369919 19967593

[ref52] Travassos B. Araújo D. Davids K. (2018). Is futsal a donor sport for football? Exploiting complementarity for early diversification in talent development. Sci. Med. Footb. 2, 66–70. doi: 10.1080/24733938.2017.1390322

[ref53] Vaeyens R. Lenoir M. Williams A. M. Mazyn L. Philippaerts R. M. (2007a). The Effects of Task Constraints on Visual Search Behavior and Decision-Making Skill in Youth Soccer Players. J. Sport Exerc. Psychol. 29, 147–169. doi: 10.1123/jsep.29.2.147, 17568064

[ref54] Vaeyens R. Lenoir M. Williams A. M. Philippaerts R. M. (2007b). Mechanisms Underpinning Successful Decision Making in Skilled Youth Soccer Players: An Analysis of Visual Search Behaviors. J. Mot. Behav. 39, 395–408. doi: 10.3200/JMBR.39.5.395-408, 17827116

[ref55] Vaeyens R. Lenoir M. Williams A. M. Philippaerts R. M. (2008). Talent identification and development programmes in sport: current models and future directions. Sports Med. 38, 703–714. doi: 10.2165/00007256-200838090-00001 18712939

[ref56] Verbeek J. Van Der Steen S. Van Yperen N. W. Den Hartigh R. J. R. (2025). What do we currently know about the development of talent? A systematic review in the soccer context. Int. Rev. Sport Exerc. Psychol. 18, 758–780. doi: 10.1080/1750984X.2023.2283874

[ref57] Ward P. Hodges N. J. Starkes J. L. Williams M. A. (2007). The road to excellence: deliberate practice and the development of expertise. High Abil. Stud. 18, 119–153. doi: 10.1080/13598130701709715

[ref58] Wells G. Shea B. O’connell D. Peterson J. Welch V. Losos M. . (2014). Newcastle-Ottawa Quality Assessment Scale Cohort Studies. Groningen, Ottava, Geneva: University of Ottawa.

[ref59] Williams A. M. Reilly T. (2000). Talent identification and development in soccer. J. Sports Sci. 18, 657–667. doi: 10.1080/02640410050120041, 11043892

[ref60] Yiannaki C. Carling C. Collins D. (2018). Could futsal hold the key to developing the next generation of youth soccer players? Sci. Med. Footb. 2, 71–74. doi: 10.1080/24733938.2017.1332422

[ref61] Zibung M. Conzelmann A. (2013). The role of specialisation in the promotion of young football talents: A person-oriented study. Eur. J. Sport Sci. 13, 452–460. doi: 10.1080/17461391.2012.749947, 24050461

